# BarH-like homeobox 2 represses the transcription of keratin 16 and affects Ras signaling pathway to suppress nasopharyngeal carcinoma progression

**DOI:** 10.1080/21655979.2022.2026549

**Published:** 2022-01-17

**Authors:** Zhibing Lu, Hui Peng, Ruijuan Li, Xinyan Xu, Jiyong Peng

**Affiliations:** Department of Oncology, Jiangxi Pingxiang People’s Hospital, Pingxiang, P.R. China

**Keywords:** Nasopharyngeal carcinoma, BARX2, KRT16, the Ras signaling pathway, proliferation

## Abstract

Nasopharyngeal carcinoma (NPC) refers to a malignancy initiating from the superior mucosal epithelium of the nasopharynx. Optimal therapies for NPC are still needed. In this investigation, we attempted to explore whether BarH-like homeobox 2 (BARX2), a well-known tumor suppressor, had anti-cancer properties on NPC, and the possible mechanisms. After searching for NPC-related databases, we determined BARX2 as one of the core genes in NPC. The results of RT-qPCR and immunohistochemistry or Western blot demonstrated that BARX2 was reduced in NPC patients and cells. Ectopic expression of BARX2 reverted the malignant phenotype of NPC cells. Mechanistically, BARX2 bound to the keratin 16 (KRT16) promoter to downregulate its expression. In addition, BARX2 was found to reduce the phosphorylation levels of MEK and ERK. Further KRT16 upregulation in cells overexpressing BARX2 promoted malignant aggressiveness of C666-1 and HNE3 cells and activated the Ras signaling pathway. BARX2 inhibited the growth and metastasis of tumors and suppressed the Ras signaling pathway *in vivo*. In conclusion, our findings indicate that BARX2 reverts malignant phenotypes of NPC cells by downregulating KRT16 in a Ras-dependent fashion. BARX2 might act as a possible therapeutic regulator for NPC.

## Introduction

Nasopharyngeal carcinoma (NPC) is characterized by distinct geographical distribution and is predominantly prevalent in east and southeast Asia [1]. Treatment regimens include radiotherapy and/or chemotherapy, and surgical excision is typically the last option for advanced and metastatic NPC, considering the closeness of nasopharynx to brain stem cell area, main blood vessels, as well as nerves [2]. Currently, the etiology of NPC comprises of three aspects: environmental factors, genetic susceptibility and Epstein-Barr virus infection, and the rise of oncogenes and the decline in tumor suppressors are the major contributors to tumorigenesis [3]. Yet, our understanding of the mechanism of NPC development are still rudimentary. Henceforth, probing the mechanisms underlying the progression in NPC is critical.

BarH-like homeobox 2 (BARX2) lies in 11q24-q25 and encodes af 254-amino acid homeodomain transcription factor [4]. As a regulator of cell adhesion and cytoskeleton remodeling [5], the reduced expression of BARX2 has been implicated in hepatocellular carcinoma and is related to poor prognosis [6]. Besides, BARX2 expression exhibits an inverse correlation with advanced tumor, node, metastases stage and a high level of Ki67 in patients with non-small cell lung carcinoma [7]. In addition, BARX2 and estrogen receptor-a bind to alternative estrogen receptor-a gene promoters in breast cancer [8]. Nevertheless, little is known concerning BARX2 in NPC. Keratin 16 (KRT16) has been identified to be expressed in the epidermis and cells of stratified squamous epithelia, ductal luminal cells and in secretory cells of human eccrine sweat glands [9]. Moreover, the role of keratins has also been appreciated in tumorigenesis and metastasis [10]. Interestingly, KRT16 was found to be activated in lung adenocarcinoma cells by the transcription factor AP-2 alpha, which contributed to the tumorigenicity [11]. Therefore, we wondered whether KRT16 was also overexpressed in NPC, and if so, its transcription was regulated by the transcription factor BARX2. The Ras/MEK/ERK signaling cascade is of great importance for inter- and intra-cellular communication, which controls basic cell functions, including growth, survival, and differentiation [12]. Silencing of KRT16 suppresses keratinocyte proliferation in psoriasis via blocking the ERK signaling pathway [13]. However, whether the similar interaction between KRT16 and the ERK signaling pathway works in cancer, especially in NPC, is scarcely understood.

In the current research, we hypothesized that BARX2 downregulates KRT16 expression to modulate the Ras signaling pathway and that BARX2 is linked to the suppression of NPC carcinogenesis. Our findings demonstrated that BARX2 reduced NPC progression via the KRT16/Ras axis. These results may provide new insights for the understanding of BARX2 in NPC pathophysiology.

## Materials and methods

### Patients and tissue samples

Thirty NPC tissues and corresponding adjacent tissues were harvested from patients who underwent surgery at Jiangxi Pingxiang People’s Hospital from March 2019 to March 2020. All patients did not receive preoperative chemotherapy or radiotherapy, and the tissue was stored at −80°C immediately after surgery. This study had approval from the Ethics Committee of Jiangxi Pingxiang People’s Hospital and performed in line with the *Declaration of Helsinki*. Written informed consent was acquired from each eligible respondent.

### Cell culture, plasmid construction and transfection

A normal human nasopharyngeal epithelial cell line NP69 was acquired from Sigma-Aldrich Chemical Company (St Louis, MO, USA). NPC cell lines, including CNE1, C666-1, HNE1, and HNE3 were from Shanghai Huiying Biotechnology Co., Ltd. (Shanghai, China). All cells were grown in Roswell Park Memorial Institute (RPMI)-1640 (Thermo Fisher Scientific Inc., Waltham, MA, USA) medium plus 10% FBS, 100 U/mL penicillin and 100 μg/mL streptomycin at 37°C and 5% CO_2_.

Plasmids, including overexpression (oe)-BARX2, siRNA targeting BARX2 (si-BARX2), oe-KRT16 and their respective controls were from GenePharma (Shanghai, China). NPC cells (1 × 10^5^) were grown in complete culture medium in 24-well plates for 24 h before transfection. We transfected the constructs into NPC cells using Lipofectamine 2000 (Thermo Fisher Scientific) and cultured them in serum-free RPMI-1640 medium for 2 d. The cells with stable expression were obtained by incubating the transfected cells with 1 μg/mL puromycin for 36 h.

### RT-qPCR

Total RNA was isolated from tissues and cells with the help of TRIzol (Thermo Fisher Scientific), and total RNA concentration was assessed using a biophotometer (Eppendorf, Hauppauge, NY, USA). RNA was reverse-transcribed into cDNA using a PrimeScript™ RT reagent Kit (Takara Holdings Inc., Kyoto, Japan) as per the manufacturer’s protocol, and the PCR system was configured using a TaqMan™ Fast Advanced Master Mix kit (Thermo Fisher Scientific). Gene expression was quantified by RT-qPCR on an Applied Biosystems 7500 Fast Real-Time PCR system (Applied Biosystems, Inc., Foster City, CA, USA). The primers were: BARX2, forward 5′-TTCGGTAGCTTTCACGTCCG-3′ and reverse 5′-ATTTCCACTCGTGCCATCCA-3′; KRT16, forward 5′-AAAGGAACCGCCCCAAATCT-3′ and reverse 5′-GTATCTCTGTGCCCGGACTG-3′; GAPDH (endogenous control) forward 5′- TTGTCAAGCTCGTTTCTTGGT −3′ and reverse 5′-CCTAGTCTCCATGGTCTCACT-3′.

### Western blot assay

The cells were lysed with radio immunoprecipitation assay buffer (Thermo Fisher scientific) spiked with protease inhibitors. Equal amounts of proteins were subjected to sodium dodecyl sulfate-polyacrylamide gel electrophoresis and transferred to NC membranes (Millipore Corp, Billerica, MA, USA). Membranes were sealed with TBST containing 5% skim milk for 60 min at room temperature and hybridized with primary antibodies overnight at 4°C. Next, the membranes were re-hybridized with the secondary antibody horseradish peroxidase (HRP)-coupled goat anti-rabbit IgG H&L (1:2,000, ab205718, Abcam, Cambridge, UK) or goat anti-mouse IgG H&L (1:2,000, ab6789, Abcam) for 1 h at ambient temperature, followed by the development. The bands were exposed and detected using a Bio-Rad ChemiDoc XRS+ imaging system (Bio-Rad, Inc., Hercules, CA, USA). Primary antibodies involved GAPDH (1:10,000, ab181602, Abcam), BARX2 (1:2,000, GTX40128, GeneTex, Inc., Alton Pkwy Irvine, CA, USA), KRT16 (1:5,000, ab76416, Abcam), p-MEK1 (1:1,000, # 9127S, Cell Signaling Technologies, Beverly, MA, USA), MEK1 (1:1,000, #12671S, Cell Signaling Technologies), p-ERK1/2 (1:1,000, ab201015, Abcam), ERK1/2 (1:10,000, ab184699, Abcam), Ki67 (1:1000, PA5-114,437, Thermo Fisher Scientific), and PCNA(1:1000, ab92552, Abcam).

### Immunohistochemistry

The isolated tumor tissues were fixed with 4% paraformaldehyde for 1 d, embedded in paraffin, and sliced at 4 μm. The sections were separated with xylene, followed by rehydration using gradient ethanol. Then, antigen extraction was performed with 10 mM citrate buffer. The tissue sections were then incubated in 3% H_2_O_2_ for 10 min and sealed with normal goat serum blocking solution for 20 min at room temperature. The sections were incubated with the primary antibodies to BARX2 (1:200, MA1-27,164, Thermo Fisher scientific), Ki67 (1:500, ab15580, Abcam), KRT16 (1:500, ab76416, Abcam), p-MEK1 (1:1000, ab129431, Abcam), p-ERK1/2 (1:1000, ab201015, Abcam) overnight at 4°C and with HRP-coupled secondary antibody goat anti-rabbit IgG H&L (1:2,000, ab205718, Abcam) or goat anti-mouse IgG H&L (1:2,000, ab6789, Abcam) for 60 min at ambient temperature. After that, the sections were stained with the peroxidase substrate diaminobenzidine (Roche Diagnostics, Indianapolis, IN, USA) for 20 min and counter-stained with hematoxylin for 1 min. The sections were paraffin-embedded after routine dehydration. Staining was observed and captured with a Leica microscope (Leica, Bannockburn, IL, USA), and brownish granules were regarded as positive expression. Analysis was performed on ImageJ, and gene expression was measured by optical density value.

### Cell counting kit-8 (CCK-8) assay

CCK-8 (Beyotime, Shanghai, China) was applied to measure the proliferative capacity of cells according to a previous study [14]. The cells were plated in 96-well culture plates at 5 × 10^3^ cells per well. After incubation to the indicated times (0 h, 24 h, 48 h, 72 h, and 96 h), 10 μL CCK-8 was supplemented to each well. The incubation continued for 2 h, and the optical density value at 450 nm was assessed by a microplate reader (Bio-Rad), and the cell growth curve was plotted based on the data obtained.

### Wound healing assay

The cells were plated into a 6-well plate (2 × 10^5^ cells/well) and cultured for 1 d as per a previous study [15]. The medium was removed when the cells reached a 90% confluence. A single wound was generated by scratching of the confluent cell monolayer using a sterile 10-μL pipette. The cells were incubated for 2 d and then observed under an inverted microscope (Olympus, Tokyo, Japan). Image J software was used to measure the 48-h migration rate of the cells.

### Transwell assay

The invasiveness of the cells was assayed using 24-well Transwell plates (Corning Costar, Cambridge, MA, USA) pre-coated with Matrigel (BD Biosciences, San Jose, CA, USA) according to a previous study [14]. The cells (1 × 10^5^) in serum-free medium were added to the apical chamber, and then the basolateral chamber was supplemented with cell culture medium containing serum. The incubation then continued in a 5% CO_2_, 37°C incubator for 1 d. The cells were fixed with 4% paraformaldehyde and stained with 0.5% crystal violet at ambient temperature (both for 10 min). Finally, crystal violet-positive cells were counted in six random fields under an inverted phase contrast microscope (Olympus), and the average number of invasive cells per field was assessed.

### Flow cytometry

Apoptosis of cells was detected using the Annexin V-fluorescein isothiocyanate (FITC)/propidium iodide (PI) Apoptosis Detection Kit (Thermo Fisher scientific) as per the manufacturer’s instructions. The cells (1 × 10^5^) were seeded into a 96-well plate and detached with trypsin and centrifuged. Afterward, the cells were incubated with 5 μL Annexin V-FITC for 5 min and with 5 μL PI for 10 min. Apoptosis rates were detected on a flow cytometer (BD Biosciences), and Flow Jo software (Becton, Dickinson and Company, Franklin Lakes, NJ, USA) was used for data analysis and graphing.

### Terminal deoxynucleotidyl transferase (TdT)-mediated 2’-Deoxyuridine 5’-Triphosphate (dUTP) nick end labeling (TUNEL)

Apoptosis was detected by a TUNEL Apoptosis Detection Kit (Beyotime). Briefly, the NPC cells (1 × 10^5^) were fixed with 4% paraformaldehyde for 30 min and subsequently permeabilized by 0.3% Triton X-100 at room temperature for 5 min. The cells were incubated with 50 μL TUNEL assay solution for 60 min at 37°C in the dark, followed by counter-staining of nuclei by incubating the cells with DAPI for 10 min at 37°C in the dark. The cells were observed by a fluorescence microscope, and the positive rate of TUENL staining (green) was calculated.

### Luciferase promoter assay

The potential binding sequence of the KRT16 promoter to BARX2 was downloaded from JASPAR (http://jaspar.genereg.net/), and the binding region was amplified using PCR and cloned into the pGL3 vector (Promega Corporation, Madison, WI, USA) to construct luciferase reporter vectors for Promoter. Promoter luciferase reporter vector was co-transfected with oe-NC or oe-BARX2 into C666-1 and HNE3 cells using Lipofectamine 2000 (Thermo Fisher Scientific). Forty-eight h after transfection, luciferase activities were examined using a luciferase assay system (Promega).

### Chromatin immunoprecipitation (ChIP)

ChIP was implemented using an EZ-Magna ChIP kit (Millipore) as described by a previous study [16]. The cells were fixed with 4% paraformaldehyde at ambient temperature for 10 min and treated with ultrasound for 2 h. The supernatant was harvested after a 5-min centrifugation at 13,000 rpm at 4°C, and incubated overnight at 4°C with antibodies to BARX2 (1:100, sc-53,177, Santa Cruz Biotechnology Inc., Santa Cruz, CA, USA) or normal mouse IgG (1:100, sc-2025, Santa Cruz), respectively. After precipitation for 0.5 h, the supernatant was discarded by centrifugation. The DNA was de-crosslinked at 65°C overnight, recovered and purified by phenol/chloroform extraction. Finally, the enrichment ability of the precipitates for the KRT16 promoter was analyzed by qPCR.

### Animal experiments

Twenty 3–4-week-old BALB/c female nude mice (15.8 ± 2.3 g, Vital River, Beijing, China) were housed in a temperature- (25°C) and humidity-controlled (45%) room on a 12/12 h light/dark cycle with ad libitum access to chow and drinking water. The mice were randomly divided into oe-NC or oe-BARX2 groups (n = 10). All animal experiments were ratified by the Ethics Committee of Jiangxi Pingxiang People’s Hospital and conducted in line with the Guide for the Care and Use of Laboratory Animals issued by NIH (Bethesda, Maryland, USA).

Five nude mice in each group were randomly selected for *in vivo* tumor growth experiments. HNE3 cells (1 × 10^6^) were diluted into 200 μL phosphate-buffered saline + 200 μL Matrigel (BD Biosciences) under sterile conditions and subcutaneously injected into anesthetized nude mice to establish a xenograft model. Tumor volume = (a*b^2^)/2 (a is the longest diameter, b is the shortest diameter) was measured once a week after injection. After 28 days, 1% pentobarbital sodium (150 mg/kg) was administered intraperitoneally to euthanize nude mice, and the tumors were weighed.

The remaining five nude mice in each group were used for *in vivo* lung metastasis experiments. HNE3 cells (1 × 10^6^) were injected into nude mice via tail vein. After 5 weeks, 1% sodium pentobarbital (150 mg/kg) was administered intraperitoneally to euthanize nude mice, and the lungs were dissected for histological exanimation. Four consecutive sections were obtained from paraffin-embedded tissue samples. The sections were stained with hematoxylin and eosin, and the area of tumor infiltration was analyzed under a light microscope.

### Statistics

All statistical analyses were implemented using GraphPad Prism 8.02 (GraphPad, San Diego, CA, USA). The data are presented as the means ± SD and analyzed using a *t* test or one-way or two-way ANOVA with Tukey’s post-hoc analysis. For cell experiments, technical triplicates were evaluated in at least three independent experiments. The Pearson’s correlation coefficient was then used to analyze the correlation between the two variables. A difference was considered significant if the *p*-value was less than 0.05.

## Results

Through bioinformatics analysis, we screened for BARX2, which was significantly poorly expressed in NPC tissues, and we hypothesized that it played a tumor suppressor role in NPC, which was verified by a series of *in vitro* and *in vivo* experiments. In further validation, we confirmed the mechanism of action of BARX2 in NPC, which blocked the Ras signaling pathway through transcriptional repression of KRT16, thus inhibiting the growth and metastasis of NPC.

### BARX2 is poorly expressed in NPC tissues

**Figure 1.**
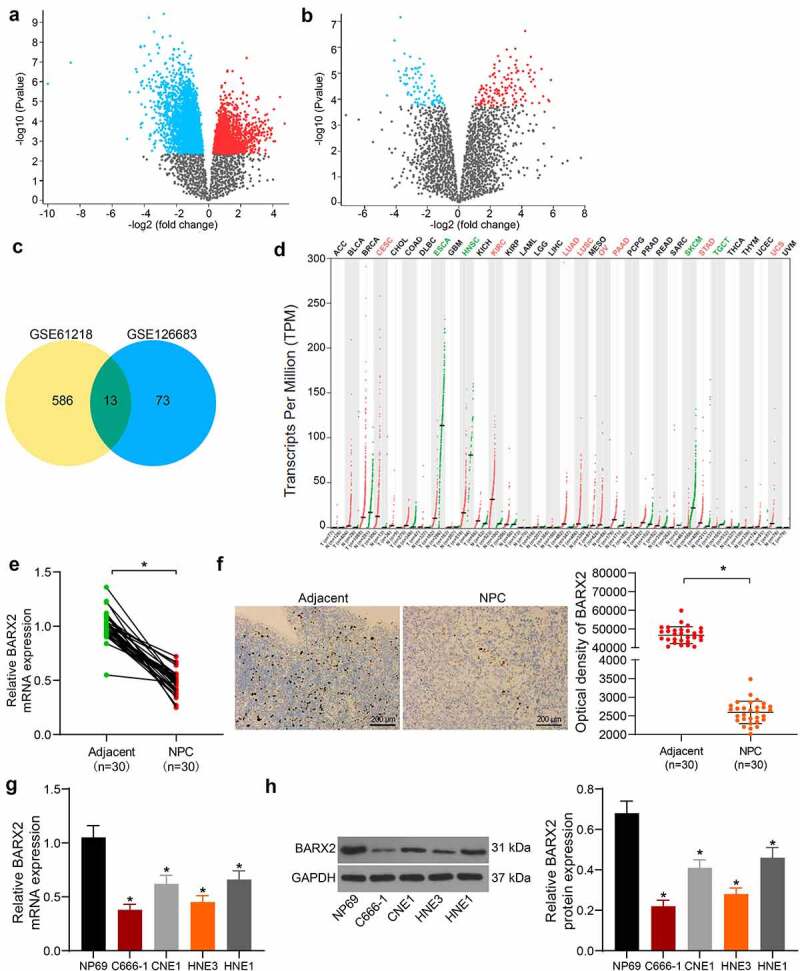
BARX2 is reduced in NPC tissues. (a-b) 586 and 73 differentially expressed genes were obtained by screening in GSE61218 (a) and GSE126683 (b) databases, respectively. (c) 13 differentially expressed genes were found in the intersection of two databases by Venn diagram. (d) expression of BARX2 in various tumor tissues from GEPIA database. (e) expression of BARX2 in collected NPC tissues and their adjacent tissues by RT-qPCR. (f) BARX2 expression in the collected NPC tissues and their adjacent tissues examined using immunohistochemical staining. (g) expression of BARX2 in normal nasopharyngeal epithelial cells NP69 and NPC cells CNE1, C666-1, HNE1, HNE3 by RT-qPCR. (h) BARX2 protein expression in normal nasopharyngeal epithelial cells NP69 and NPC cell lines CNE1, C666-1, HNE1, HNE3 examined using Western blot. Results are shown as mean ± SD, and the data were represented by three independent experiments. The paired t-test and one-way ANOVA was utilized for statistical analysis. *p < 0.05 *vs* adjacent tissues or NP69 cells.

The GSE61218 database comprises epithelium from 10 NPC patients and 6 normal healthy nasopharyngeal tissue specimens. The GSE126683 database includes 3 pairs of NPC and normal nasopharynx tissues. By setting Log FoldChange > 2.0 and Adj. *p* value < 0.05 as screening thresholds, we obtained differentially expressed genes from the two databases and plotted the volcano maps (Figure 1a, b). The screened genes were subjected to overlapping by a Venn diagram, and 13 genes were found to be differentially expressed in both databases (Figure 1c). Subsequently, we further queried from the GEPIA (http://gepia.cancer-pku.cn/) database and found significant decline in BARX2 expression in several cancers (Figure 1d). The BARX2 mRNA expression in 30 NPC tissues and adjacent tissues was detected by RT-qPCR. It was observed that its expression was much lower in the NPC tissues than those in the adjacent tissues (Figure 1e). Immunohistochemical staining presented that the BARX2 protein expression was also reduced in NPC tissues (Figure 1f). We further analyzed the expression of BARX2 in normal nasopharyngeal epithelial cells NP69 and NPC cells CNE1, C666-1, HNE1, HNE3. The mRNA and protein expression of BARX2 in NPC cells was appreciably lower than that in NP69 cells (Figure 1g, h). C666-1 and HNE3 cells with more pronounced alteration were chosen for the following studies.

### Overexpression of BARX2 impedes the growth, migration and invasion of NPC cells

**Figure 2.**
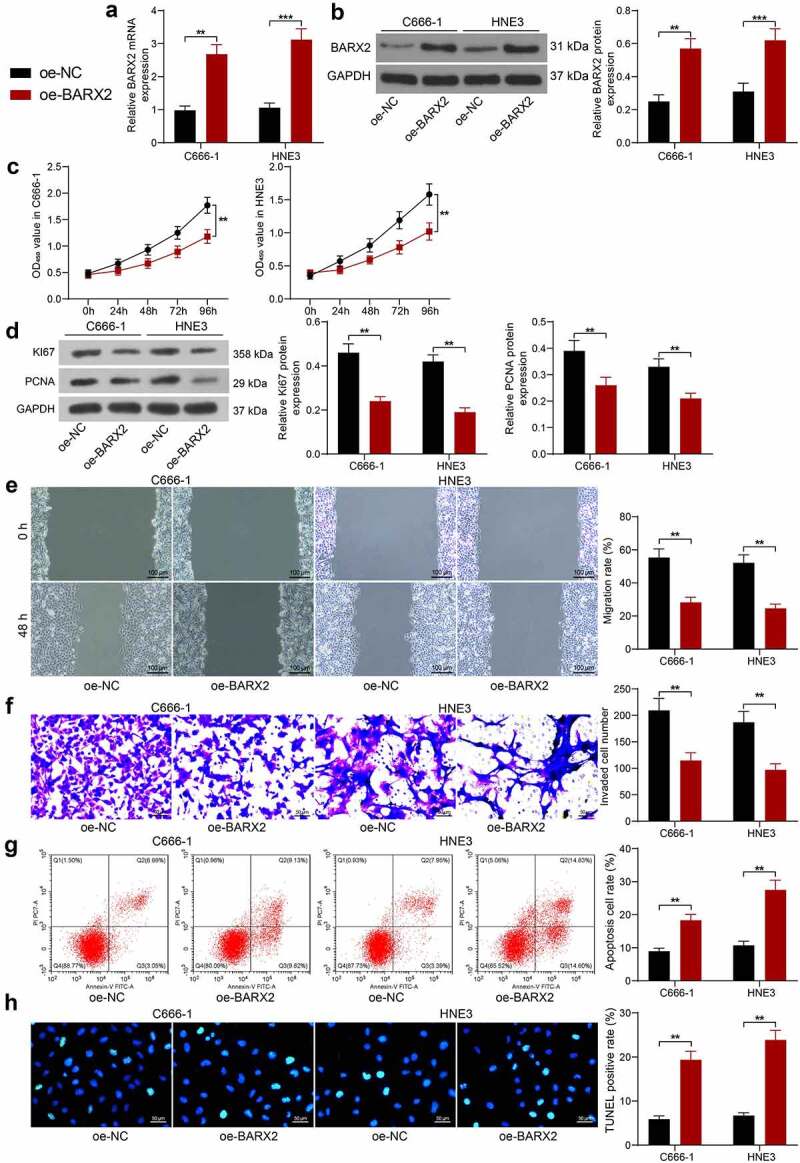
Ectopic expression of BARX2 hinders the growth, migration, and invasion of NPC cells *in vitro*. (a) BARX2 mRNA expression in C666-1 and HNE3 by RT-qPCR. (b) BARX2 protein expression in C666-1 and HNE3 by Western blot. (c) cell proliferative activity after BARX2 overexpression examined using CCK8. (d) expression of proliferation marker proteins Ki67 and PCNA after overexpression of BARX2 examined using Western blot. (e) cell migratory ability after overexpression of BARX2 measured using wound healing assay. (f) cell invasive activity after overexpression of BARX2 measured using Transwell assays. (g) cell apoptosis after overexpression of BARX2 examined using flow cytometry. (h) apoptosis rate of cells after overexpression of BARX2 examined using TUNEL. Results are shown as mean ± SD, and the data were represented by three independent experiments. The two-way ANOVA was utilized for statistical analysis. **p< 0.01, ***p< 0.001 *vs* oe-NC.

To examine the role of BARX2 in NPC cells, we upregulated BARX2 in C666-1 and HNE3 cells. The transfection efficiency was substantiated by RT-qPCR and Western blot, and the expression of both mRNA and protein of BARX2 was remarkably promoted in C666-1 and HNE3 cells (Figure 2a, b). We first assayed the cell growth using CCK-8 assay, and we observed that the growth rate of C666-1 and HNE3 was considerably reduced after overexpression of BARX2 (Figure 2c). Western blot experiments showed that overexpression of BARX2 significantly inhibited the protein expression of proliferation-associated markers Ki67 and PCNA in cells (Figure 2d). In addition, cell migration and invasion were examined by wound healing assay and Transwell invasion assay, respectively. The migration and invasion capabilities of C666-1 and HNE3 cells were appreciably inhibited after overexpression of BARX2 (Figure 2e, f). Finally, we used flow cytometry and TUNEL to assess apoptosis, and we found that the percentage of apoptosis in C666-1 and HNE3 cells was increased after overexpression of BARX2 (Figure 2g, h).

### BARX2 represses KRT16 expression at the transcriptional level

**Figure 3.**
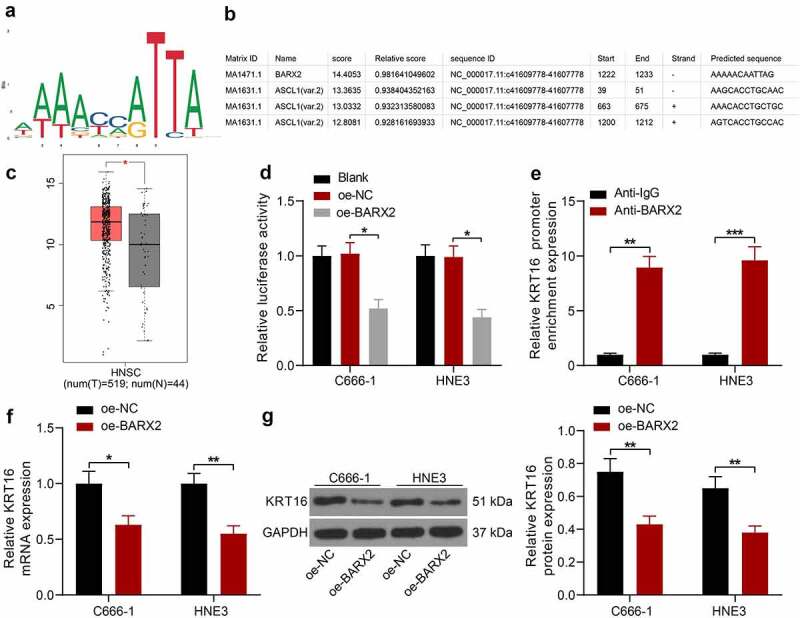
BARX2 represses the expression of KRT16 at the transcription level. (a) conserved binding sites for BARX2. (b) potential binding sites of KRT16 promoter to BARX2. (c) KRT16 expression in NPC patients queried in GEPIA. (d) the effect of BARX2 inhibition on Promoter luciferase activity examined using dual-luciferase assay. (e) the enrichment ability of anti-BARX2 on KRT16 promoter sequence measured using ChIP assay. (f-g) mRNA (f) and protein (g) expression of KRT16 in C666-1 and HNE3 cells overexpressing BARX2 by RT-qPCR and Western blot. Results are shown as mean ± SD, and the data were represented by three independent experiments. The two-way ANOVA was utilized for statistical analysis. *p < 0.05, **p< 0.01, ***p< 0.001 *vs* blank, oe-NC, or anti-IgG.

To investigate the pathway through which BARX2 acts on NPC, we obtained its conserved binding sequence (Figure 3a) in Jaspar (http://jaspar.genereg.net/) and predicted the potential binding site of the KRT16 promoter to BARX2 (Figure 3b). Through the GEPIA database, we observed that KRT16 was significantly upregulated in NPC (Figure 3c). The Promoter luciferase reporter vector was constructed by inserting the site with the highest predicted score into the luciferase reporter vector. Through dual-luciferase assays, we observed that BARX2 significantly hindered the luciferase activity of Promoter (Figure 3d). In ChIP experiments, anti-BARX2 significantly enriched the KRT16 promoter sequence compared to IgG (Figure 3e). Moreover, the mRNA and protein expression of KRT16 was significantly suppressed after we overexpressed BARX2 expression in C666-1 and HNE3 cells (Figure 3f, g). The above experiments demonstrated that BARX2 represses KRT16 transcription by binding to the KRT16 promoter.

### BARX2 impairs the Ras pathway by downregulating KRT16

**Figure 4.**
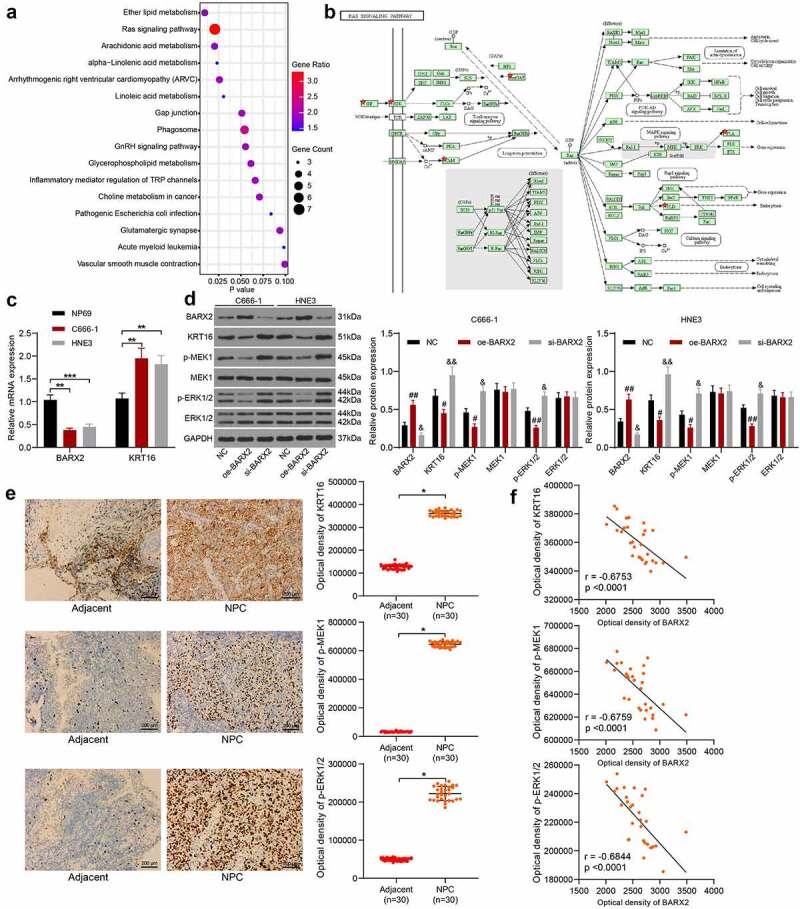
BARX2 inhibits the Ras signaling by suppressing the expression of KRT16. (a) genes with correlation coefficients larger than 0.5 with KRT16 in the TCGA database analyzed using KEGG enrichment analysis. (b) KEGG enrichment analysis of downstream proteins. (c) expression of BARX2 and KRT16 in NP69, C666-1 and HNE3 cells by RT-qPCR. (d) protein expression of BARX2, KRT16, pMEK1, MEK1, p-ERK1/2 and ERK1/2 in C666-1 and HNE3 cells examined using Western blot. (e) Immunohistochemical detection of KRT16, p-MEK1, and p-ERK1/2 expression in tissues of patients with NPC (n = 30). (f) Correlation between the expression of KRT16, p-MEK1, p-ERK1/2 and BARX2 in NPC tissues. Results are shown as mean ± SD, and the data were represented by three independent experiments. Unpaired *t* test or the two-way ANOVA was utilized for statistical analysis. **p< 0.01, ***p< 0.001 *vs* NP69 cells or adjacent tissues; ^#^*p*< 0.05, ^##^*p*< 0.01, or ^&^*p*< 0.05, ^&&^*p*< 0.01 *vs* NC.

We analyzed the genes that correlated with KRT16 (correlation score > 0.5) in TCGA database to investigate the mechanism of KRT16 in NPC and identified that the Ras signaling pathway was significantly enriched (Figure 4a), in which Raf1, MEK and ERK were enriched (Figure 4b). In comparison with normal nasopharyngeal epithelial cell NP69, C666-1 and HNE3 cells exhibited poor BARX2 expression and high KRT16 expression, as revealed by RT-qPCR (Figure 4c). The expression of BARX2, KRT16 and downstream proteins pMEK1, MEK1, p-ERK1/2 and ERK1/2 was assessed by Western blot in NPC cells after overexpression or silencing of BARX2. After overexpression of BARX2, BARX2 expression was significantly increased, while KRT16 expression and phosphorylation levels of both MEK and ERK were significantly decreased. After knockdown of BARX2, BARX2 expression was significantly decreased, whereas KRT16 expression and the phosphorylation levels of both MEK and ERK were significantly augmented (Figure 4d). The expression of KRT16, p-MEK1 and p-ERK1/2 was detected by immunohistochemistry in our collected NPC tissues, and the expression of these proteins was significantly elevated in NPC tissues (Figure 4e). Furthermore, the expression of these proteins in NPC tissues was negatively correlated with the expression of BARX2 protein (Figure 4f). The above results suggest that BARX2 blocks the Ras signaling pathway by suppressing the expression of KRT16, thereby suppressing NPC carcinogenesis.

### Upregulation of KRT16 inhibits the suppressive effect of oe-BARX2 on the malignant biological behavior of NPC cells

**Figure 5.**
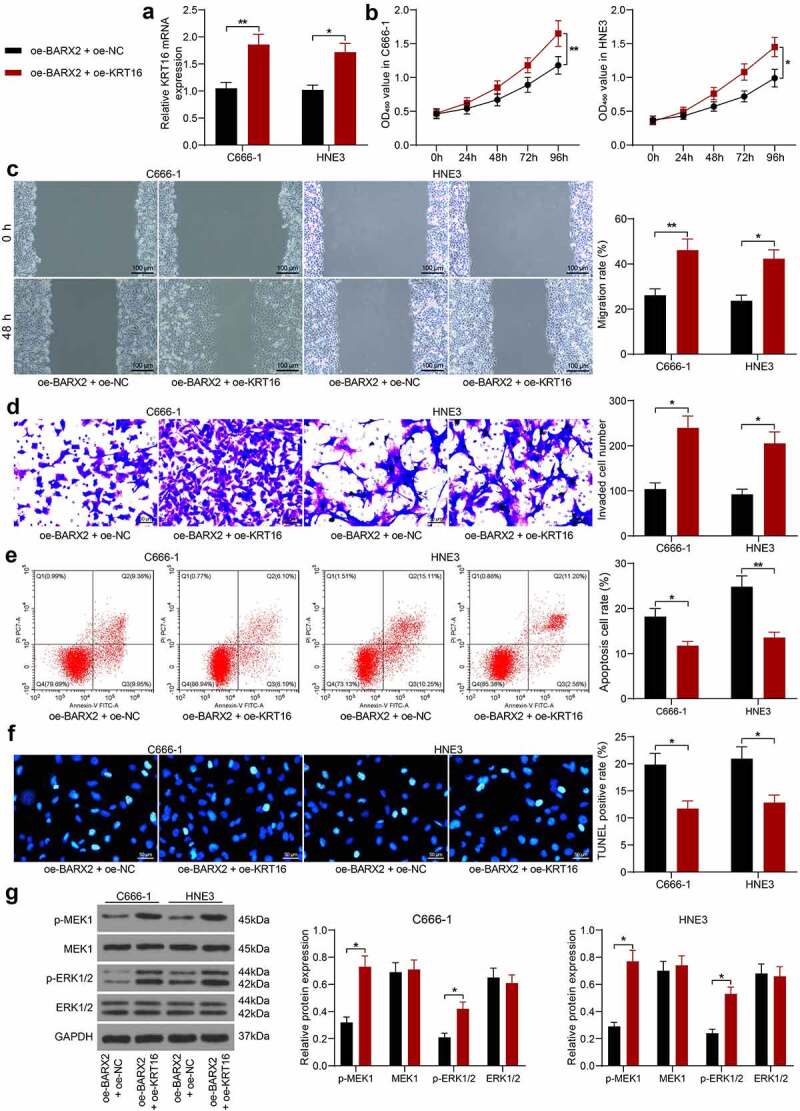
Upregulation of KRT16 significantly inhibits the role of oe-BARX2 on the malignant biological behavior of NPC cells. Overexpression of KRT16 was transfected into C666-1 and HNE3 cells overexpressing BARX2. (a) mRNA expression of KRT16 in cells by RT-qPCR. (b) cell proliferative activity examined using CCK8. (c) cell migratory ability measured using wound healing assay. (d) cell invasive activity measured using Transwell assays. (e) cell apoptosis examined using flow cytometry. (f) cell apoptosis rate examined using TUNEL. (g) protein expression of pMEK1, MEK1, p-ERK1/2 and ERK1/2 in cells after overexpression of KRT16 examined using Western blot. Results are shown as mean ± SD, and the data were represented by three independent experiments. The two-way ANOVA was utilized for statistical analysis. *p< 0.05, **p< 0.001 *vs* oe-BARX2 + oe-NC.

To corroborate the involvement of KRT16 in BARX2-mediated NPC cell malignant biological behaviors, we overexpressed KRT16 in C666-1 and HNE3 cells stably overexpressing BARX2. RT-qPCR was used to corroborate the successful construction of cell lines overexpressing KRT16 (Figure 5a). Our analysis by CCK8 assay revealed that further overexpression of KRT16 in cells overexpressing BARX2 contributed to a remarkable increase in cellular activity (Figure 5b). We used the wound healing assay to test the migration of cells and observed a significant rise in the 48-h migration rate of C666-1 and HNE3 cells after further upregulation of KRT16 (Figure 5c). A same trend as in the migration assay was observed in the invasion assay, i.e., upregulation of KRT16 reversed the effect of oe-BARX2 and elevated the invasive ability of the cells (Figure 5d). Subsequently, we used flow cytometry and TUNEL to detect apoptosis, and we found that overexpression of KRT16 contributed to a remarkable reduction in the proportion of apoptosis in C666-1 and HNE3 cells (Figure 5e, f). Western blot assay exhibited that the phosphorylation levels of both MEK and ERK were drastically increased, and the Ras signaling pathway was activated after overexpression of KRT16 (Figure 5g). The above results denote that upregulation of KRT16 expression activated the Ras pathway by elevating phosphorylation levels of MEK and ERK and inhibited the repressive effect of oe-BARX2 on the malignant biological behavior of NPC cells.

### *BARX2 constrains the growth and metastases of NPC cells* in vivo

**Figure 6.**
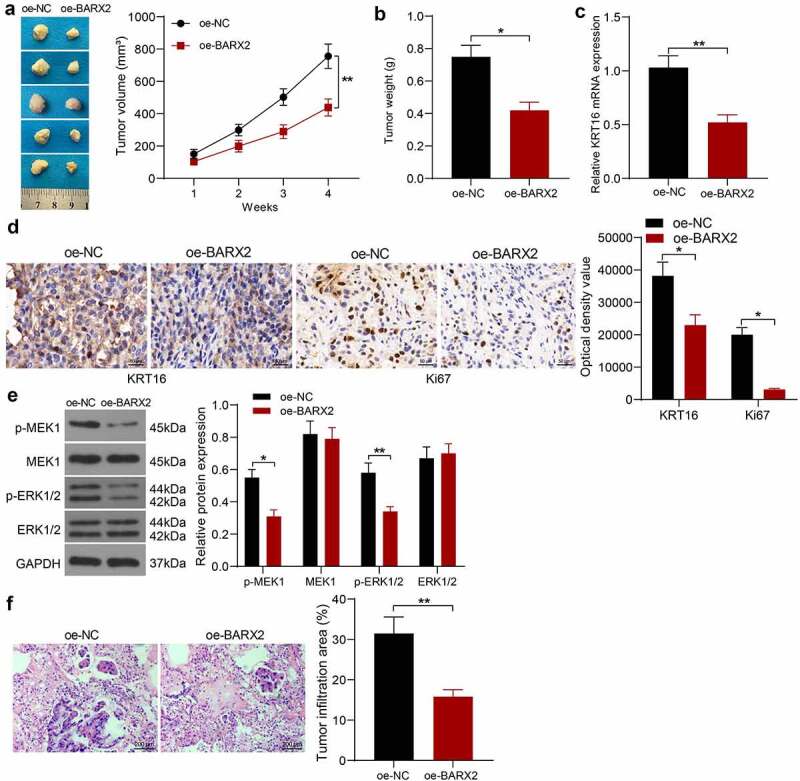
BARX2 impedes the growth and metastases of NPC cells *in vivo*. (a) changes in tumor volume in mice examined using *in vivo* tumorigenesis assay. (b) assessment of cellular tumorigenic capacity by tumor weight change. (c) mRNA expression of KRT16 in tumor tissues by RT-qPCR. (d) immunohistochemical analysis of KRT16 and ki67 in tumor tissues. (e) protein expression of pMEK1, MEK1, p-ERK1/2 and ERK1/2 in tumor tissues examined using Western blot. (f) lung infiltration of tumor cells examined using HE staining. Results are shown as mean ± SD (n = 5). The unpaired t-test and two-way ANOVA was utilized for statistical analysis. *p < 0.05, **p < 0.01 *vs* oe-NC.

We selected HNE3 cells with stable high expression of BARX2 for nude mice inoculation to probe the role of BARX2 on the growth of NPC cells *in vivo*. The tumor volumes were examined every 7 days. It was found that the growth rate of xenograft tumors formed by HNE3 cells was decreased in the presence of oe-BARX2 (Figure 6a, b). The xenograft tumors were harvested after 4 weeks, and the mRNA levels of KRT16 in the tissues were detected by RT-qPCR, which indicated that the mRNA expression of KRT16 was downregulated after BARX2 overexpression (Figure 6c). Immunohistochemical detection of KRT16 and ki67 in tumor tissues demonstrated that the expression of KRT16 and Ki67 was reduced in xenograft tumors from mice injected with oe-BARX2 (Figure 6d). Finally, Western blot assay of Ras signaling pathway activity in tumor tissues showed that the phosphorylation levels of MEK and ERK were decreased in nude mice harboring BARX2 overexpression (Figure 6e). An *in vivo* tumor metastasis model was generated by tail vein injection of tumor cells, and we observed that overexpression of BARX2 also inhibited tumor metastasis to the lung *in vivo* by HE staining (Figure 6f). It indicates that BARX2 suppresses tumorigenesis and metastasis *in vivo* by modulating the transcription of KRT16 to inhibit the Ras signaling pathway.

## Discussion

NPC, a kind of head and neck malignancy with a characteristic regional and racial prevalence, is related to Epstein-Barr virus infection and highly prone to develop regional and distant metastases [17]. In China, NPC ranked the 11^th^ among all cancers in 2008, and the trend is toward a lower rate in North and West China and a higher incidence in South and East China [18]. Consequently, there has been a strong drive to decipher the molecular mechanism underlying the progression of NPC. The data acquired from this study showed that KRT16 overexpression abrogated the BARX2-suppressed malignant phenotypes of NPC cells by modulating the Ras signaling pathway. Based on these findings, BARX2 overexpression or KRT16 silencing may show an interest of therapeutic targets in patients with NPC.

Firstly, by mining the NPC-related databases, we found out BARX2 was one of the differentially expressed genes in NPC. BARX2 represents a homeobox gene of the Bar class and is expressed in neural and craniofacial structures in the process of development [19]. Downregulation of BARX2 has been verified to predict the poor survival in patients with colorectal cancer [20]. Consistently, BARX2 expression was downregulated in the vast majority of oral squamous cell carcinoma, and this downregulation was more pronounced in tumors with advanced nodal metastasis [21]. Moreover, BARX2 was downregulated by CpG Island methylation and was linked to repressed gastric cancer cell proliferation and invasion [22]. Yet, little is known concerning its function in NPC. We found the downregulation of BARX2 was identified in both NPC tissues and cells. In addition, overexpression of BARX2 effectively inhibited NPC cell proliferation, migration, and invasion, whereas enhanced apoptosis. Under the condition of non-small-cell lung cancer, miR-942 elevated cell migration, invasion, and angiogenesis, which was reversed by BARX2 overexpression [23]. These results were in line with our findings. Furthermore, our *in vivo* evidence provided that BARX2 overexpression could delay the tumor growth and lung metastasis in NPC.

To expound the downstream mechanism, we applied bioinformatics prediction. The binding relation between BARX2 and KRT16 has been substantiated via ChIP and dual-luciferase assays. The proximal promoter of the BARX2 gene harbored binding sites for various factors including MyoD, myogenin, serum response factor, as well as myocyte enhancer factor [24]. Additionally, BARX2 is a transcription factor that modulates the transcription of cell adhesion molecules in mice [25]. However, the role of BARX2 as a transcription factor has been rarely investigated in cancers. Keratins comprise up to 85% of the protein content of differentiated epithelial cells and offer flexibility and strength by creating a framework within the cytoplasm [26]. Previously, Endo *et al*. reported that a transcription factor NRF2 could mediate the expression of KRT16 in human keratinocytes [27]. In addition, microRNA-365-3p targeted ETS homologous factor, a KRT16 transcription factor, to suppress migration, invasion, and metastasis in oral squamous cell carcinoma cells by suppressing KRT16 [28]. Our rescue assays also validated that KRT16 mitigated the repressing effects of BARX2 on NPC cell malignant phenotype.

Following the determination of KRT16 as a target of BARX2, we utilized TCGA database to screen genes with a correlation score larger than 0.5 with KRT16. The Ras signaling pathway was validated as the downstream effector of KRT16. Ras proteins could control intracellular signaling pathways involved in important cellular processes, such as cell proliferation, migration, and apoptosis, and Ras activates some pathways, including the MEK-ERK/MAPK cascade, which leads to the transcription of genes controlling various cellular mechanisms [29]. In NPC cells overexpressing BARX2, the Ras signaling pathway was significantly impaired, as evidenced by reduced phosphorylation levels of both MEK and ERK. Similarly, human circular RNA_101705 promoted the growth and invasion in renal cell carcinoma by activating the MAPK/ERK pathway [30]. More relevantly, the suppression of isoprenylcysteine carboxylmethyltransferase blocked the Ras signaling in NPC cells [31]. Mechanistically, silencing of KRT16 induced protein degradation of β5-integrin and c-Met, contributing to blockage of the downstream Src/STAT3/FAK/ERK signaling in oral squamous cell carcinoma cells [28]. Here, we observed that KRT16 reversed the effects of BARX2 and activated the Ras signaling pathway again.

## Conclusion

In conclusion, our study reveals that BARX2 suppresses KRT16 transcription, leading to Ras signaling pathway deficit to inhibit NPC cell proliferation. Based on all these observations, BARX2 may be a possible candidate against NPC. Nevertheless, more studies are still necessary in the future for its clinical application.

## Data Availability

All the data generated or analyzed during this study are included in this published article.
